# RNA interference-mediated silencing of BACE and APP attenuates the isoflurane-induced caspase activation

**DOI:** 10.1186/2045-9912-1-5

**Published:** 2011-04-28

**Authors:** Yuanlin Dong, Zhipeng Xu, Yiying Zhang, Sayre McAuliffe, Hui Wang, Xia Shen, Yun Yue, Zhongcong Xie

**Affiliations:** 1 Geriatric Anesthesia Research Unit, Department of Anesthesia, Critical Care and Pain Medicine, Massachusetts General Hospital and Harvard Medical School, Charlestown, MA 02129-2060; USA; 2 Department of Anesthesia, Beijing Chaoyang Hospital, Capital Medical University, Beijing, 100020, P.R. China; 3 Department of Anesthesiology, Shanghai Eye, Ear, Nose and Throat Hospital, Fudan University, Shanghai 200031, P.R. China

## Abstract

**Background:**

β-Amyloid protein (Aβ) has been shown to potentiate the caspase-3 activation induced by the commonly used inhalation anesthetic isoflurane. However, it is unknown whether reduction in Aβ levels can attenuate the isoflurane-induced caspase-3 activation. We therefore set out to determine the effects of RNA interference-mediated silencing of amyloid precursor protein (APP) and β-site APP-cleaving enzyme (BACE) on the levels of Aβ and the isoflurane-induced caspase-3 activation.

**Methods:**

H4 human neuroglioma cells stably transfected to express full-length human APP (H4-APP cells) were treated with small interference RNAs (siRNAs) targeted at silencing BACE and APP for 48 hours. The cells were then treated with 2% isoflurane for six hours. The levels of BACE, APP, and caspase-3 were determined using Western blot analysis. Sandwich Enzyme-linked immunosorbent assay (ELISA) was used to determine the extracellular Aβ levels in the conditioned cell culture media.

**Results:**

Here we show for the first time that treatment with BACE and APP siRNAs can decrease levels of BACE, full-length APP, and APP c-terminal fragments. Moreover, the treatment attenuates the Aβ levels and the isoflurane-induced caspase-3 activation. These results further suggest a potential role of Aβ in the isoflurane-induced caspase-3 activation such that the reduction in Aβ levels attenuates the isoflurane-induced caspase-3 activation.

**Conclusion:**

These findings will lead to more studies which aim at illustrating the underlying mechanism by which isoflurane and other anesthetics may affect Alzheimer's disease neuropathogenesis.

## Background

Alzheimer's disease (AD), one of the most common forms of dementia, affects 4.5 million Americans and costs more than $100 billion a year on direct care alone. Its impact will only increase in the coming decades. AD is an insidious and progressive neurodegenerative disorder and is characterized by global cognitive decline, robust accumulation of amyloid deposits, and neurofibrillary tangles in the brain [reviewed in [1]]. Genetic evidence, confirmed by neuropathological and biochemical findings, indicates that excessive production and/or accumulation of β-amyloid protein (Aβ) play a fundamental role in the pathology of AD [reviewed by [1,2]]. Aβ is produced from amyloid precursor protein (APP) through proteolytic processing by the aspartyl protease β-site APP-cleaving enzyme (BACE) and γ-secretase [reviewed in [3]].

Increasing evidence suggests a role for caspase activation and apoptosis in AD neuropathogenesis [[4-13], reviewed in [14,15]]. There has been debate in regards to the contribution of apoptosis to neuronal loss in AD because the apoptotic markers are rarely detected in the brain of AD patients [reviewed in [16,17]]. However, this could be due to the long duration of AD and very rapid clearance of apoptotic cells from organs. Recent studies employing antibodies that specifically recognize caspase-cleaved substrates have shown that caspase-3-cleaved-actins, caspase-3-cleaved fragments, and caspase-cleaved-APPs are present in AD patients' brains [18-31]. Western blot analysis has also revealed increased caspase-3 immunoreactivity in AD versus control brains [24,32,33]. In addition, activated caspase-6 and caspase-9 have been detected in AD brains [25,26].

An estimated 200 million patients worldwide undergo anesthesia and surgery each year [34,35]. Both surgery and anesthesia have been suggested to play a role in the progress of AD neuropathogenesis [reviewed in [36,37]] and AD. Specifically, the age of onset of AD has been reported to be inversely related to cumulative exposure to anesthesia and surgery before the age of 50 years [38], even though anesthesia and/or surgery themselves may not increase the incidence of AD [39]. Another study showed that patients having coronary artery bypass graft surgery under general anesthesia may be at increased risk for AD as compared to those having percutaneous transluminal coronary angioplasty under local anesthesia [40]. A recent retrospective population-based study has found that general anesthesia is a risk factor of AD with an adjusted odds ratio of 3.22 [41]. Moreover, cognitive dysfunction or decline occurs after anesthesia and surgery [[42-52], reviewed in 53], which is associated with impairments in daily functioning [54], dependency on government economic assistance [52], and increased morbidity and mortality [[42,55], reviewed in [56]]. However, opposing findings also exist [57-59]. Therefore, more clinical studies, which will define the role of anesthesia and/or surgery in AD and in postoperative cognitive dysfunction or decline, are necessary [60].

Given the fact that adequately powered prospective human studies will take many years to conduct and analyze, it is equally important to perform animal and *in vitro *studies, which will complement ongoing human studies, e.g., by establishing a mechanistic hypothesis. Several studies have shown that the commonly used inhalation anesthetic isoflurane may induce caspase activation, apoptosis, Aβ oligomerization and accumulation, neuroinflammation, tau protein hyperphosphorylation, mitochondrial dysfunction, and impairment of learning and memory [[60-69], reviewed in [36,37]]. However, the underlying mechanisms of these effects remain largely to be determined. Our studies in cultured cells have shown that exogenerously administrated Aβ into the cell culture media can potentiate the isoflurane-induced caspase activation and apoptosis, which may induce further rounds of apoptosis and Aβ generation [70]. In the present studies, we set out to determine the effects of RNA interference (RNAi)-mediated silencing of BACE and APP on Aβ levels and the isoflurane-induced caspase activaion in cultured cells to further elucidate the potential association of Aβ accumulation and the isoflurane-induced caspase-3 activation.

## Methods

### Cell lines

We employed H4 human neuroglioma cells stably transfected to express full-length human APP (H4-APP cells) in the experiments. We used H4-APP cells for the easy measurement of Aβ levels in the conditioned cell culture media as we did in the previous studies [65,70,71]. The cells were cultured in Dulbecco's modified Eagle's medium (high glucose) containing 9% heat-inactivated fetal calf serum, 100 units/ml penicillin, 100 g/ml streptomycin, and 2 mM L-glutamine and was supplemented with 20 g/ml G418.

### RNAi studies

RNAi-mediated silencing of BACE and APP experiments were similar to those in our previous studies [72-76]. In order to avoid off-target effects of RNAi, we employed two sets of small interference RNAs (siRNAs) aimed at silencing of BACE (1^st ^set: 3'GCAAGGAGUACAACUAUGAUU; 2^nd ^set: 3'GGAGGGAGCAUGAUCAUUGUU) and APP (1^st ^set: 3' GGUGGGCGGUGUUGUCAUA; 2^nd ^set: 3' GGUUCUGGGUUGACAAAUA). These siRNAs and control siRNA (3'UAGCGACUAAACACAUCAAUU) were obtained from Dharmacon (Lafayette, CO). siRNAs were transfected into cells using electroporation (AMAXA, Gaithersburg, MD) as described by Xie et al [75]. Briefly, we mixed 1 million cells, 100 ul AMAXA electroporation transfection solution and 10 ul 20 uM siRNA together, then we employed C-9 program in an AMAXA electroporation device for cell transfection. The transfected cells were then placed in one of the six-well plates containing 1.5 ml cell culture media. The BACE, APP, or control siRNA-pretreated cells were then exposed to the isoflurane treatment 48 hours later.

### Isoflurane treatment

The isoflurane treatment was similar to those in our previous studies [65,70,71]. We chose 2% isoflurane (air component: 2% isoflurane, 5% CO_2_, 21% O_2 _and balanced nitrogen) in the studies based on our previous studies [65,70,71]. The control condition included 5% CO_2 _plus 21% O_2 _(air component: 5% CO_2_, 21% O_2 _and balanced nitrogen), which did not affect caspase-3 activation or Aβ levels (Data not shown). The delivery of gases was similar to that described in our previous studies [65,70]. Briefly, 21% O_2_, 5% CO_2_, and 2% isoflurane were delivered from an anesthesia machine to a sealed plastic box (airtight chamber) in a 37 degree C incubator containing six-well plates seeded with one million cells in a 1.5 ml cell culture media. The Datex infrared gas analyzer (Puritan-Bennett, Tewksbury, MA) was used to continuously monitor the concentrations of CO_2_, O_2_, and isoflurane that were delivered.

### Lysis of cells and protein amount quantification

The pellets of the cells were detergent-extracted on ice using an immunoprecipitation buffer (10 mM Tris-HCl, pH 7.4, 150 mM NaCl, 2 mM EDTA, 0.5% Nonidet P-40) plus protease inhibitors (1 g/ml aprotinin, 1 g/ml leupeptin, 1 g/ml pepstatin A). The lysates were collected and centrifuged at 12,000 × g for 10 minutes, and then were quantified for total protein levels using the bicinchoninic acid protein assay kit (Pierce, Iselin, NJ).

### Western blot analysis

The cells were harvested at the end of the experiments and were subjected to Western blot analyses using the methods described by Xie et al. [70]. BACE antibody (1:1,000 dilution; Abcam, Cambridge, MA) was used to recognize BACE (65 kDa). Antibody A8717 (1:1,000 dilution; Sigma, St. Louis, MO) was used to recognize FL-APP (110 kDa) and APP-CTFs (10 to 12 kDa). A caspase-3 antibody (1:1,000 dilution; Cell Signaling Technology, Inc. Beverly, MA) was used to recognize the caspase-3 fragment (17-20 kDa), which results from cleavage at the asparate position 175, and FL-caspase-3 (35 - 40 kDa). An antibody to the non-targeted protein β-Actin (42 kDa, 1:5,000, Sigma) was used to control for loading differences in total protein amounts. Each band in the Western blot represents an independent experiment. We have averaged the results from three to six independent experiments. The intensity of signals in each Western blot was analyzed using the National Institute of Health image program (National Institute of Health Image 1.62, Bethesda, MD). We quantified the Western blots using two steps. First, we used levels of β-Actin to normalize (e.g., determine the ratio of the amount of FL-caspase-3 to the amount of β-Actin) the levels of FL-caspase-3, caspase-3 fragment, BACE, FL-APP, and APP-CTFs to control for any loading differences in total protein amounts. Second, we presented changes in the levels of BACE, FL-APP, APP-CTFs, and caspase-3 in the treated cells as percentages of those in cells from the control condition.

### Quantification of Aβ using Sandwich ELISA assay

Secreted Aβ in the conditioned culture media was measured with a Sandwich ELISA assay by using an Aβ measurement kit (Invitrogen, Carlsbad, CA) as described by Xie et al. [75]. Specifically, 96-well plates were coated with mouse monoclonal antibodies (mAb) specific to Aβ_40 _(2G3) or Aβ_42 _(21F12). Following blocking with Block Ace, wells were incubated overnight at 4°C with test samples of conditioned cell culture media, and then an anti-Aβ (β-A-HR1) antibody conjugated to horseradish peroxidase was added. Plates were then developed with TMB reagent and well absorbance was measured at 450 nm. Aβ levels in test samples were determined by comparison with the signal from unconditioned media spiked with known quantities of Aβ_40 _and Aβ_42_.

### Statistics

Given the presence of background caspase-3 activation, Aβ, BACE, FL-APP, and APP-CTFs in the cells cultured in serum free media, we did not use absolute values to describe their changes. Instead, these changes were presented as percentages of those from the control group. For example, one hundred percent of caspase-3 activation refers to the control level for the purpose of comparison to experimental conditions. Data were expressed as mean ± S.D.. The number of samples varied from three to six, and the samples were normally distributed. We used a two-tailed t-test to compare the difference between the control siRNA and BACE or APP siRNA, and the control condition and isoflurane treatment. P-values less than 0.05 (*) and 0.01 (** or ##) were considered statistically significant.

## Results and discussion

### RNAi-mediated silencing of BACE attenuates the isoflurane-induced caspase-3 activation

We previously reported that the commonly used inhalation anesthetic isoflurane can induce caspase activation and apoptosis *in vitro *[65,70,71] and *in vivo *[64]. However, the underlying mechanisms of these effects remain largely to be determined. Specifically, Aβ has been shown to potentiate the isoflurane-induced caspase-3 activation in H4 naïve cells, but it is largely unknown whether reduction in the levels of Aβ can decrease the isoflurane-induced caspase-3 activation in the cultured cells. BACE is the enzyme for Aβ generation and APP is the precursor of Aβ. Decreases in the levels of BACE and APP could lead to reduction in Aβ levels [3]. We therefore set out to assess the effects of RNAi-mediated silencing of BACE and APP on the levels of Aβ and the isoflurane-induced caspase-3 activation in H4-APP cells.

**Figure 1:**
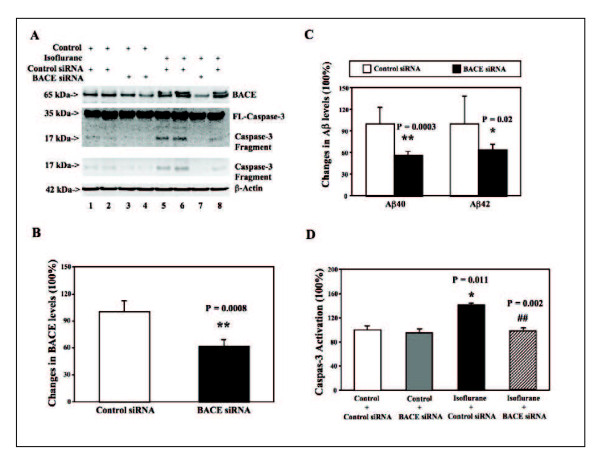
**Effects of RNAi-mediated silencing of BACE on Aβ levels and caspase-3 activation in H4-APP cells**. **A**. Treatment of BACE siRNA (lanes 3, 4 and 7) decreases the levels of BACE as compared to control siRNA (lanes 1, 2, 5, 6 and 8) in the Western blotting analysis. Isoflurane treatment (lanes 5 and 6) induces caspase-3 activation as compared to control condition (lanes 1 and 2). The BACE siRNA treatment alone (lanes 3 and 4) does not induce caspase-3 activation. However, the BACE siRNA treatment (lane 7) attenuates the isoflurane-induced caspase-3 activation (lanes 5, 6 and 8) as compared to control siRNA treatment (lanes 5, 6 and 8). **B**. Quantification of the Western blots shows that BACE siRNA treatment (black bar) decreases the levels of BACE (** P = 0.0008) as compared to control siRNA treatment (white bar). **C**. BACE siRNA treatment (black bar) reduces the levels of Aβ40 (left panel, ** P = 0.0003) and Aβ42 (right panel, * P = 0.02) as compared to control siRNA treatment (white bar). **D**. Quantification of the Western blots shows that isoflurane (black bar, * P = 0.011) induces caspase-3 activation as compared to control condition (white bar). BACE siRNA treatment (net bar, ## P = 0.002) attenuates the isoflurane-induced caspase-3 activation as compared to control siRNA plus isoflurane treatment (black bar).

The H4-APP cells were treated with control or BACE siRNA for 48 hours before the treatment with 2% isoflurane for six hours. The cells were harvested at the end of the experiment and were subjected to Western blot analysis. BACE immunoblotting showed that the BACE siRNA treatment decreased BACE levels as compared to the control siRNA treatment (Figure 1A). The quantification of the Western blots illustrated that BACE siRNA treatment significantly decreased BACE levels as compared to control siRNA: 100% versus 57% (Figure 1B). These findings suggest that the treatment with BACE siRNA, which targets at reducing mRNA levels of BACE, was able to reduce the protein levels of BACE in the current experiment. Next, we were able to show that the BACE siRNA treatment decreased the levels of both Aβ40 (100% versus 55%) and Aβ42 (100% versus 63%) (Figure 1C). These results suggested that the BACE siRNA was able to reduce Aβ generation by decreasing the levels of BACE, the enzyme of Aβ generation.

As expected, the caspase-3 immunoblotting showed that the treatment with 2% isoflurane (lanes 5, 6 and 8) for six hours induced caspase-3 activation, as evidenced by increased ratios of cleaved (activated) caspase-3 fragment (17 kDa) to full-length (FL) caspase-3 (35 - 40 kDa), compared with control condition (lanes 1 and 2). Finally, we were able to show that the BACE siRNA treatment (lane 7) attenuated the isoflurane-induced caspase-3 activation (lanes 5, 6 and 8) (Figure 1A). The quantification of the Western blots showed that the isoflurane treatment (black bar) induced caspase-3 activation as compared to control condition (white bar): 100% versus 148%. The BACE siRNA treatment alone (gray bar) did not induce caspase activation. However, the BACE siRNA treatment attenuated the isoflurane-induced caspase-3 activation (net bar) (Figure 1D): 148% versus 103%. These results illustrate that reduction in BACE levels, via RNAi-mediated silencing of BACE, may lead to the reduction of Aβ levels and the attenuation of the isoflurane-induced caspase-3 activation.

### RNAi-mediated silencing of APP attenuates the isoflurane-induced caspase-3 activation

Given the findings that reduction in the levels of both BACE and Aβ is associated with the attenuation of the isoflurane-induced caspase-3 activation, next, we would like to know whether other methods to reduce Aβ levels can also lead to the attenuation of the isoflurane-induced caspase-3 activation. Therefore, we set out to determine the effects of RNAi-mediated silencing of APP, the precursor of Aβ, on the levels of APP and Aβ, and on the isoflurane-induced caspase-3 activation.

**Figure 2:**
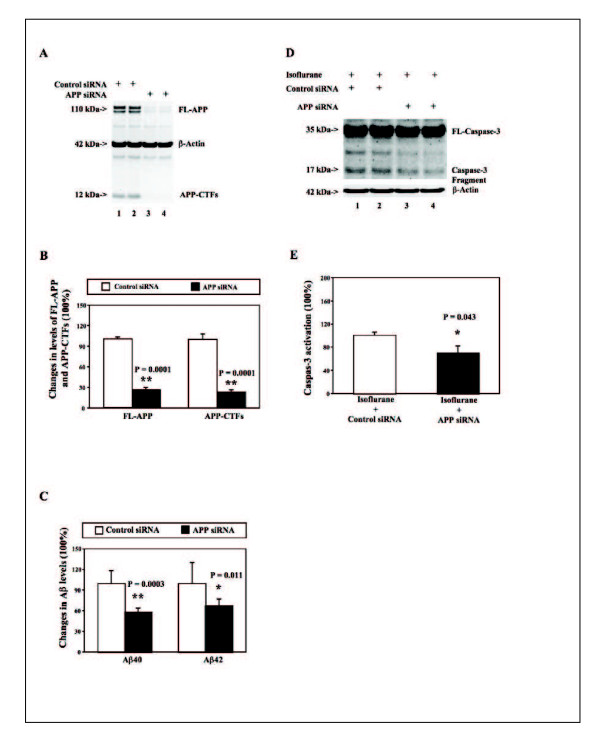
**Effects of RNAi-mediated silencing of APP on Aβ levels and caspase-3 activation in H4-APP cells**. **A**. Treatment of APP siRNA (lanes 3 and 4) decreases the levels of FL-APP and APP-CTFs as compared to control siRNA (lanes 1 and 2) in the Western blotting analysis. **B**. Quantification of the Western blots shows that APP siRNA treatment (black bar) decreases the levels of FL-APP (left panel, ** P = 0.0001) and APP-CTFs (right panel, ** P = 0.0001) as compared to control siRNA treatment (white bar). **C**. APP siRNA treatment (black bar) reduces the levels of Aβ40 (left panel, ** P = 0.0003) and Aβ42 (right panel, * P = 0.011) as compared to control siRNA treatment (white bar). **D**. APP siRNA treatment (lanes 3 and 4) attenuates the caspase-3 activation induced by isoflurane as compared to the control siRNA treatment (lanes 1 and 2) in the Western blotting analysis. **E**. Quantification of the Western blots shows that APP siRNA treatment (black bar, * P = 0.043) reduces the isoflurane-induced caspase-3 activation as compared to control siRNA plus isoflurane treatment (white bar).

The H4-APP cells were treated with control or APP siRNA for 48 hours before the treatment with 2% isoflurane for six hours. The cells were harvested at the end of the experiment and were subjected to Western blot analysis. The APP immunoblotting showed that the APP siRNA treatment (lanes 3 and 4) decreased the levels of FL-APP and APP-CTFs as compared to the control siRNA treatment (lanes 1 and 2) (Figure 2A). The quantification of the Western blots showed that the APP siRNA treatment (black bar) decreased the levels of FL-APP (left panel, 100% versus 26%) and APP-CTFs (right panel, 100% versus 23%) as compared to control siRNA treatment (white bar). These results suggest that the RNAi-mediated silencing of APP was able to reduce the levels of APP in the H4-APP cells in the current experiment.

Next, we were able to show that the APP siRNA treatment reduced the levels of both Aβ40 (left panel, 100% versus 58%) and Aβ42 (right panel, 100% versus 66%). Finally, the caspase-3 immunoblotting showed that the APP siRNA treatment (lanes 3 and 4) decreased the isoflurane-induced caspase-3 activation as compared to the control siRNA treatment (lanes 1 and 2) (Figure 2D). The quantification of the Western blots showed that the APP siRNA treatment (black bar) decreased the isoflurane-induced caspase-3 activation as compared to control siRNA treatment (white bar): 100% versus 64%. These results illustrated that the reduction in the levels of Aβ and APP, resulting from RNAi-mediated silencing of APP, may also lead to the attenuation of isoflurane-induced caspase-3 activation.

Taken together, these findings suggest that there is an association between the Aβ levels and the isoflurane-induced caspase-3 activation, specifically, the reduction of Aβ levels, resulted from RNAi-mediated silencing of either BACE or APP, can lead to the attenuation of the isoflurane-induced caspase-3 activation.

Our previous studies have shown that the commonly used inhalation anesthetic isoflurane can induce caspase-3 activation and apoptosis [64,65,70,71]. However, the underlying mechanism remains unclear and is an important question in the field of anesthesia neurotoxicity research. The previous studies in H4 naïve and H4-APP cells have shown that the isoflurane-induced caspase-3 activation and apoptosis can enhance levels of BACE and γ-secretase, which promote APP processing and increase Aβ generation [70]. Moreover, Aβ can potentiate the isoflurane-induced caspase-3 activation, leading to further rounds of apoptosis [70]. However, it is largely unknown whether reduction in Aβ levels can attenuate the isoflurane-induced caspase-3 activation. Therefore, we set out to assess the effects of RNAi-mediated silencing of APP, the precursor of Aβ, and BACE, the enzyme of Aβ generation, on Aβ levels and on the isoflurane-induced caspase-3 activation in H4-APP cells.

First, we have found that RNAi-mediated silencing of BACE can decrease BACE levels. These results suggest that the BACE siRNA-induced reduction in BACE mRNA levels can successfully decrease the protein levels of BACE in the current experiment. Then, we have found that there is a decrease in Aβ levels following the BACE siRNA treatment. Finally, the BACE siRNA treatment attenuates the isoflurane-induced caspase-3 activation in the H4-APP cells. These results have suggested that decreased Aβ levels by the RNAi-mediated silencing of BACE may lead to the attenuation of the isoflurane-induced caspase-3 activation. These results further support our previous findings that isoflurane may induce a vicious cycle of caspase-3 activation/apoptosis and Aβ accumulation [70].

The double bands for BACE in Figure 1A could be the isoforms of BACE. It is also possible that isoflurane induces a post-translational modification of BACE (e.g., phosphorylation). However, the RNAi of BACE decreases both bands of BACE, thus these findings still support the conclusion of current study that RNAi-mediated silencing of BACE can lead to a reduction in Aβ levels and an attenuation of the isoflurane-induced caspase-3 activation. As the key enzyme that initiates the formation of Aβ, BACE is a prerequisite for the generation of Aβ, which gives rise to cerebrovascular and parenchymal amyloid plaque in the brain of AD patients. Thus, it is important to identify these double bands following the isoflurane treatment in the future studies.

Previous in vivo studies have shown that a 50% reduction in BACE1 levels causes only a 12% decrease in Aβ levels in heterozygous BACE1 gene knock-out mice [77]. However, our current in vitro studies have illustrated that a 43% reduction in BACE levels, following the BACE siRNA treatment, led to a 45% and a 37% reduction in the levels of Aβ40 and Aβ42, respectively. It is largely unknown why there is a difference between the in vitro and in vivo findings in the Aβ levels. The possible explanations include the difference in the methods and experimental variability.

Decreased levels of BACE in heterozygous (BACE1+/-) mice can lead to improvement of hippocampus-independent and -dependent form of memory deficits in the AD animal model [78,79]. Isoflurane has been shown to induce learning and memory impairment [62,80,81]. Our future studies, therefore, will include assessing the effects of isoflurane on learning and memory in heterozygous (BACE1+/-) mice to further determine the role of BACE and Aβ in the anesthesia associated neurotoxicity.

Next, we have further demonstrated the potential association of Aβ accumulation and isoflurane-induced caspase-3 activation by showing that RNAi-mediated silencing of APP can decrease the levels of FL-APP, APP-CTFs, Aβ, and finally the isoflurane-induced caspase-3 activation. These findings have suggested that the reduction in Aβ levels by decreasing the levels of its precursor i.e., APP, can also lead to the attenuation in the isoflurane-induced caspase-3 activation.

Isoflurane has been reported to induce caspase activation and apoptosis [64,65,70,76,82], [reviewed in [36,37]]. However, different findings do exist [83-93]. The reason for the different effects of isoflurane is largely unknown. Some studies have suggested that isoflurane may have a concentration and/or time-dependent dual effect (protective versus toxic) [94-96]. However, given the findings that increases and decreases in Aβ levels can either potentiate [70] or attenuate (current findings) the isoflurane-induced caspase-3 activation, respectively, it is possible that isoflurane may have different effects on caspase-3 activation and apoptosis when different Aβ levels are presented. Additional studies will be needed to further test this hypothesis by determining the effects of different concentrations of exogenously administrated Aβ on the isoflurane-induced caspase-3 activation and apoptosis *in vitro *and *in vivo*.

## Conclusion

In conclusion, we have found that RNAi-mediated silencing of either BACE or APP can lead to a reduction in Aβ levels as well as an attenuation in the isoflurane-induced caspase-3 activation. These results have further supported our previous findings that isoflurane induces caspase activation and apoptosis, which lead to Aβ accumulation. Aβ will then cause further rounds of caspase activation and apoptosis [70]. We would like to emphasize that although our current findings and the results from other studies have suggested that isoflurane may promote AD neuropathogenesis, it is still premature to conclude that isoflurane is toxic to use in patients. The *in vivo *relevance of these effects of isoflurane in humans remains largely to be determined. Nevertheless, our current findings should lead to additional studies to determine the potential effects of anesthetics on AD neuropathogenesis and the underlying mechanisms. These efforts will ultimately help facilitating the design of safer anesthetics and improved anesthesia care for patients, especially elderly individuals and patients with AD.

## List of Abbreviation

AD: Alzheimer's disease; APP: amyloid β precursor protein; BACE: β-site amyloid precursor protein-cleaving enzyme; Aβ: β-amyloid protein; CTFs: c-terminal fragments.

## Competing interests

The authors declare that they have no competing interests.

## Authors' contributions

YD: Acquisition of data. ZX: Acquisition of data, Analysis and interpretation of data, Critical revision of the manuscript for important intellectual content. YZ: Acquisition of data, Critical revision of the manuscript for important intellectual content. SM: Critical revision of the manuscript for important intellectual content. HW: Administrative, technical, and material support. X S: Administrative, technical, and material support. YY: Obtained funding, Critical revision of the manuscript for important intellectual content. ZX: Obtained funding, Study concept and design, Analysis and interpretation of data, Drafting of the manuscript, Critical revision of the manuscript for important intellectual content, Study supervision. All authors read and have approved the manuscript.

## Authors' information

Geriatric Anesthesia Research Unit, Department of Anesthesia, Critical Care and Pain Medicine, Massachusetts General Hospital and Harvard Medical School (Dong, Z., Xu, Z., Zhang, Y., McAuliffe, S., Wang, H., Shen, X., and Xie, Z.).

Department of Anesthesia, Beijing Chaoyang Hospital, Capital Medical University, Beijing, P.R. China (Wang, H. and Yue, Y.).

Department of Anesthesiology, Shanghai Eye, Ear, Nose and Throat Hospital, Fudan University, Shanghai 200031, P.R. China (Shen, X.).
